# Enhanced Sol–Gel Route to Obtain a Highly Transparent and Conductive Aluminum-Doped Zinc Oxide Thin Film

**DOI:** 10.3390/ma12111744

**Published:** 2019-05-29

**Authors:** Mohammad Hossein Nateq, Riccardo Ceccato

**Affiliations:** Department of Industrial Engineering, University of Trento, Via Sommarive 9, 38123 Trento, Italy; riccardo.ceccato@unitn.it

**Keywords:** sol–gel, Al-doped ZnO, hydrolysis, thin film, transparent conductors, resistivity, UV-Vis-NIR spectroscopy, figure of merit

## Abstract

The electrical and optical properties of sol–gel derived aluminum-doped zinc oxide thin films containing 2 at.% Al were investigated considering the modifying effects of (1) increasing the sol H_2_O content and (2) a thermal treatment procedure with a high-temperature approach followed by an additional heat-treatment step under a reducing atmosphere. According to the results obtained via the TG-DTA analysis, FT-IR spectroscopy, X-ray diffraction technique, and four-point probe resistivity measurements, it is argued that in the modified sample, the sol hydrolysis, decomposition of the deposited gel, and crystallization of grains result in grains of larger crystallite size in the range of 20 to 30 nm and a stronger c-axis preferred orientation with slightly less microstrain. The obtained morphology and grain-boundary characteristics result in improved conductivity considering the resistivity value below 6 mΩ·cm. A detailed investigation of the samples’ optical properties, in terms of analyzing their absorption and dispersion behaviors through UV-Vis-NIR spectroscopy, support our reasoning for the increase of the mobility, and to a lesser extent the concentration of charge carriers, while causing only a slight degradation of optical transmittance down to nearly 80%. Hence, an enhanced performance as a transparent conducting film is claimed for the modified sample by comparing the figure-of-merit values.

## 1. Introduction

Having high optical transmission together with low electrical resistivity is a unique feature exclusive to transparent conducting films (TCFs), affording them a number of specific applications in optoelectronic devices such as liquid crystal displays, light-emitting diodes, the electromagnetic shielding of cathode-ray tubes, functional glasses, photoelectrochemical cells, and sensors [1].

Among the limited number of materials possessing such a property, the most widely utilized are thin films of certain wide band-gap n-type semiconducting oxides such as In_2_O_3_, SnO_2_, and ZnO, which are known as transparent conducting oxides (TCOs). Here, the non-stoichiometric excess of cations or oxygen vacancies can provide a considerable number of charge carriers, causing an intrinsically moderate but usually not sufficient electrical conductivity, which is significantly boosted when properly doped with appropriate elements [2]. However, due to the trade-off between electrical conductivity and optical transmission [3], the quality of a TCO is evaluated by a factor called figure-of-merit (FoM) to consider both properties simultaneously.

The most commonly used TCO material is indium tin oxide (ITO), since it offers the highest FoM value [4]. However, the global shortage and increasing price of indium has triggered attempts to enhance the properties of more affordable TCOs, especially aluminum-doped ZnO (AZO) [5,6]. On an industrial scale, the deposition of AZO and other TCO films is usually performed through expensive vacuum-based technologies such as sputtering to obtain high-quality films. Another approach is using wet deposition techniques such as the sol–gel method, which provides a considerably more affordable way without the necessity of applying high temperature or vacuum conditions [2]. They are also quite suitable for samples with sizable or non-flat surfaces such as tubes [7]. In spite of such advantages, the sol–gel derived films are often porous, of lower quality, and have less FoM values than those of sputtered films [8], which necessitates further investigation to improve the sol–gel procedure. To date, the solution-based methods have been considered highly promising in the inkjet printing of TCOs for low-cost printed electronics and solar cells [9].

In the sol–gel synthesis of thin films, nanocrystalline or amorphous as-deposited layers transform into the crystalline state by the post-deposition crystallization, throughout which the crystal orientation and grain-boundary characteristics develop. These microstructural features affect the optical and electrical properties, and hence, the FoM value. Accordingly, controlling the film crystallization is a crucial step in the sol–gel synthesis of TCOs. Based on the barrier model theory proposed by Seto on the electrical properties of polycrystalline silicon films [10], and its expansion to AZO structures [11], Ohyama [12] and Nishio [13] concluded that AZO shows higher conductivity in the case of having a highly oriented microstructure toward the c-axis of the zincite structure, compared with less oriented or granular microstructures. Considering that the conductivity σ is directly proportional with the concentration N and mobility μ of charge carriers as σ ~ N·μ, a higher concentration results in less optical transmission, while the effect of the mobility value on transmission is insignificant [14]. Accordingly, a highly oriented microstructure shows higher conductivity because the mobility of charge carriers is enhanced by grain-boundary scattering being less effective. Thus, higher conductivity is obtained while the optical transmission is unaffected, which mean a higher FoM value. Heretofore, many attempts have been devoted to improving the performance of the sol–gel derived AZO thin films through studying how microstructural features are influenced by processing parameters, including solvent and stabilizer types [15,16,17,18], precursor and doping concentrations [15,16,17], deposition conditions [12,16], the drying temperature [12,18,19], and finally, annealing cycles and atmosphere [12,18,20,21]. However, to the best of our knowledge, these studies have focused on each parameter independently, and there is no investigation to include the optimum values for all the processing parameters. Moreover, a recently proposed modification to improve the films’ conductivity value is studied more profoundly here. Accordingly [22], by adding water to the coating sol, the subsequent change in the hydrolysis reaction leads to a decrease in the resistivity of the final AZO thin film. This study attempts to clarify the reasons for such an observation, and the results contribute to the sol–gel deposition of AZO thin films with higher FoM values, and therefore, superior optoelectrical performance.

## 2. Materials and Methods

### 2.1. Sol Preparation

**Figure 1:**
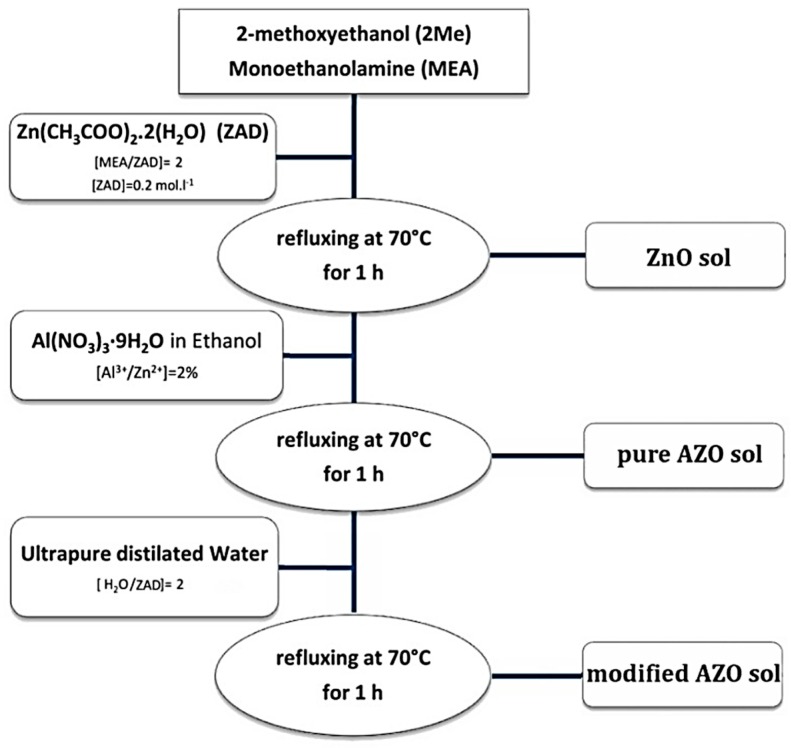
The flowchart summarizing the sol’s preparation steps.

Figure 1 shows the sol preparation steps. Zinc acetate dihydrate (ZAD), Zn(CH_3_COO)_2_·2(H_2_O), was first dissolved at room temperature in a round-bottom twin-neck flask containing 2-methoxyethanol (2-Me) as the solvent and monoethanolamine (MEA) as the stabilizer agent. The concentration of ZAD was 0.2 mol·L^−1^ and the molar ratio of MEA to ZAD was [MEA/ZAD] = 2. Then, it was heated under reflux for 1 h at 70 °C to yield a clear and homogeneous solution. A part of the obtained solution was removed and transferred into another flask and kept under stirring at room temperature to serve as the ZnO sol henceforth. For the remaining part, the doping solution, which was ethanolic solution of aluminum nitrate nonahydrate, Al(NO_3_)_3_·9H_2_O, was added drop by drop. The molar ratio of the dopant, [Al^3+^/Zn^2+^], was precisely selected: 2%. Then, it was left under reflux for a further 1 h at 70 °C to get a clear and quite pale yellowish solution. A part of the newly obtained solution was removed once again and transferred into another flask and kept under stirring at room temperature to serve as the pure AZO sol thereafter. The third part of the solution was modified via altering the hydrolysis reaction, through adding drop by drop a precise amount of ultrapure distilled water. The molar ratio of additional water to ZAD was [H_2_O/ZAD] = 2. Then, it was left under reflux for a further 1 h at 70 °C, whereby a slightly stronger yellowish solution was obtained. It was kept under stirring at room temperature to serve as the modified AZO sol in future. All three flasks were dynamically aged for 72 h to yield a proper viscosity and a suitable colloidal condition. A pH measurement was performed for the sols afterwards.

### 2.2. Gel and Powder Processing

The remaining part of the main flask was used to prepare AZO gel and powder. The gel was obtained by keeping a portion of the solution in 80 °C for almost two weeks insofar as that a bright brownish sticky soft substance was obtained. The remaining sol was kept under reflux at 70 °C for 24 h; whereby white AZO powder precipitated. The powder was collected after washing three times with ethanol and centrifuging at 6000 rpm for 20 min, followed by annealing at 600 °C for 2 h.

### 2.3. Substrates Preparation

The specimens were produced in two different groups—the Group A over soda-lime and the Group B over silica substrates—all with the dimension of 2.5 × 3.8 cm^2^. The substrates’ preparation started by washing them by a phosphate-free liquid detergent of neutral pH and 5 min of ultrasonic cleaning in acetone, ethanol, and distilled water, respectively. Next, in order to have highly hydrophilic surfaces with outstanding wettability, a ternary treating procedure was performed, which was 10 min rinsing in piranha solution, a 5% solution of ammonium hydroxide, and a 5% solution of hydrochloric acid, followed by rinsing in distilled water and drying with compressed nitrogen blow.

### 2.4. Film Deposition and Annealing

A home-made dip-coating apparatus was used for the film deposition. All the depositions were performed under identical conditions with a withdrawal speed of 2.5 cm·min^−1^. The dipping chamber was kept at room temperature and filled with N_2_ to have a dry atmosphere during the depositions and the subsequent drying. Before dipping the substrates, the coating sols were filtered through Millipore Millex-FG hydrophobic Teflon filters of 0.2 μm. Then, they were warmed up to roughly 40 °C in order to ensure homogeneous viscosity, better substrates coverage, and in particular, an enhanced solvent evaporation after the withdrawal. The latter improves the layer thickness uniformity, and therefore avoids the formation of pale whitish haze-looking zones on the coated areas [23]. After the withdrawal, samples were kept for 2 min in the dry atmosphere of the dipping chamber to start the drying and gelation reaction. It was followed by a 5-min intermediate heat treatment through introducing the Group A and Group B samples to an electrical furnace heated up to 275 °C and 430 °C, respectively. The layer deposition cycle was repeated 15 times for each sample to obtain an adequate thickness. At the end, the final heat treatment was performed in static air for 1 h at 500 °C and 600 °C for Group A and Group B, respectively. An additional heat treatment was also performed only for Group B samples in Ar flow at 600 °C for 1 h followed by another 1 hour at 400 °C in Ar/H_2_ flow containing 3% hydrogen. The thermal treatment details and thermal history of the samples is summarized in Table 1.

### 2.5. Characterization

DTA-TG measurements were performed on a Netzsch STA-409 instrument (NETZSCH Group, Selb, Germany), in the range of 20–700 °C with a heating rate of 10 °C·min^−1^, in static air; alumina crucibles for both reference and sample were used. Baseline correction for the DTA curve and buoyancy correction for TG were carried out by means of an empty crucibles experiment. FT-IR analysis was performed with a Nicolet Avatar 330 instrument, in transmittance mode; powdered gel was dissolved in KBr in order to get a pellet. The same instrument was further utilized to measure the IR transmittance of the deposited thin films as well. The usual adopted scan conditions were: 4000–400 cm^−1^ as the wavenumber range, and the number of scans was equal to 64 with a resolution of 4 cm^−1^. XRD spectra were acquired on a Rigaku DMax-III D diffractometer (Rigaku Corporation, Tokyo, Japan), employing a CuKα radiation (λ = 0.154056 nm) and a graphite monochromator in the diffracted beam. Asymmetric scattering configuration was adopted for film measurements, with typical parameter values: scan range in 2θ: 10° to 50°; counting time: 10 s; sample interval: 0.1°; and the incidence angle set at 1°. For the evaluation of crystallite dimensions, line profile analysis on the fitted peaks was performed with MAUD software [24]. To measure the sheet resistance of the obtained films in square geometry, the four-point probe method was applied at room temperature. The films’ thicknesses were determined through spectroscopic ellipsometry by a HORIBA-UVISEL ellipsometer (HORIBA Ltd., Kyoto, Japan) equipped with DeltaPsi2 software. Finally, the normal transmittance and near-normal reflectance spectra were obtained by a UV-Vis-NIR spectrophotometer JASCO V570 (JASCO International Co. Ltd., Tokyo, Japan) at room temperature in the range of 300–2500 nm with the resolution of 2 nm.

## 3. Results

**Figure 2:**
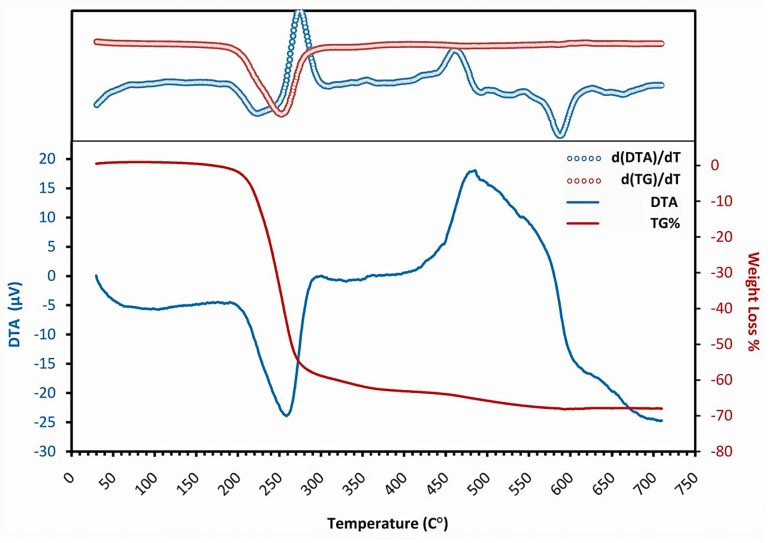
The TG-DTA graph of the dried gel. The derivatives are plotted in the upper part.

Figure 2 shows the TG-DTA and derivative curves of the dried AZO gel heated in static air with the rate of 10 °C·min^−1^. It indicates that the weight loss occurs in two different steps. The first one is observed as a sharp drop down to nearly 55% in the range of 195 to 275 °C, corresponding to the endothermic peak centered at 250 °C. During the other step, a mild weight loss starts at around 275 °C, and is continuous in a long temperature range up to 590 °C, comprising about another 15% weight loss. In the DTA graph, it associates with a long-range exothermic pattern resulting from the overlap of successive exothermic reactions, from which sharp ones centered at around 300 °C and 485 °C are identified. No other weight loss is observed after 600 °C.

**Figure 3:**
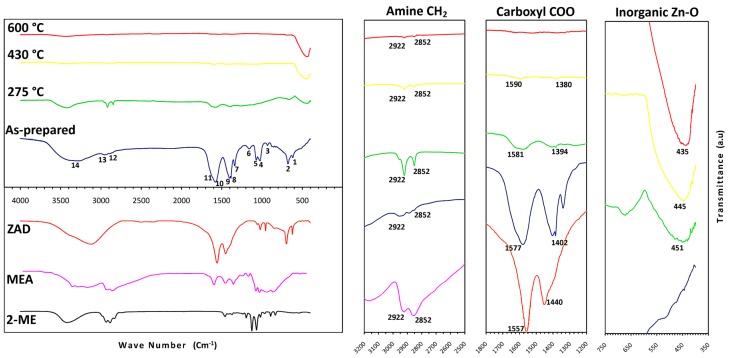
The IR spectra of the as-prepared aluminum-doped ZnO (AZO) gel and its thermal evolution at 275 °C, 430 °C, and 600 °C (upper left), in addition to the data of initial compounds, zinc acetate dihydrate (ZAD), monoethanolamine (MEA) and 2-methoxyethanol (2-ME) (lower left). The spectrum concerning 500 °C is not shown here, since it is quite similar to the one at 600 °C. Magnified parts of the spectra regarding the CH_2_ bond, COO bond, and Zn–O bond (right). The initial compounds’ data were taken from Bio-Rad’s IR spectral databases.

Figure 3 illustrates the IR spectra of the dried AZO gel and its thermal evolution at 275 °C, 430 °C, and 600 °C, in addition to ZAD, MEA, and 2-ME data as the initial compounds. The spectrum concerning 500 °C is not shown here, since it is quite similar to the one at 600 °C. Higher magnifications of the graph are also included from the spectral range of 400 to 700 cm^−1^, 1200 to 1800 cm^−1^, and 2500 to 3200 cm^−1^, corresponding to the IR spectrum range of Zn-O, carboxyl COO, and amine CH_2_ bonds, respectively.

For the dried gel, a superposition of ZAD and MEA peaks is observed, mainly including CH_3_, COO, and OH absorption peaks of ZAD in the range of 1000 to 1100 cm^−1^, 1400 to 1600 cm^−1^, and 3200 to 3400 cm^−1^, respectively, as well as CH_2_ deformations and CH_2_ stretching peaks of MEA in the range of 1300 to 1500 cm^−1^ and 2800 to 3000 cm^−1^, respectively. The 2-Me peaks must be overlapped by other peaks, since the long-term evaporation allows just a little amount of 2-Me to remain in the sticky gel. Distinct absorption peaks of the as-prepared gel are numbered as one to 14 under the spectrum, and their wavenumbers as well as brief explanations regarding the bonds and vibration modes of the constituent peaks are listed in Table 2.

While no evidence is detected for the Zn–O bond in the gel, after heat treatment at 275 °C, together with a considerable reduction of all the organic bonds, an absorption peak of the metal oxide bond appears at around 451 cm^−1^. It is related to the stretching vibration of the Zn–O bond in tetrahedral coordination [25]; whereas we cannot clearly draw conclusions about the presence of the Zn–O bond in octahedral coordination that shows an absorption peak at around 670 cm^−1^ [25], due to overlapping with the α and π bonds of COO peaks of ZAD. The trend continues for the treated gels at 430 and 600 °C with the increasing peak intensity of the Zn–O bond in tetrahedral coordination and the diminishing of remaining organic bonds, while the formation of the Zn–O bond in octahedral coordination seems to be totally insignificant. In the spectra of treated gel at 500 and 600 °C, only a sharp Zn–O bond peak is detected, with a minor trace of CH_2_ and OH bonds.

**Figure 4:**
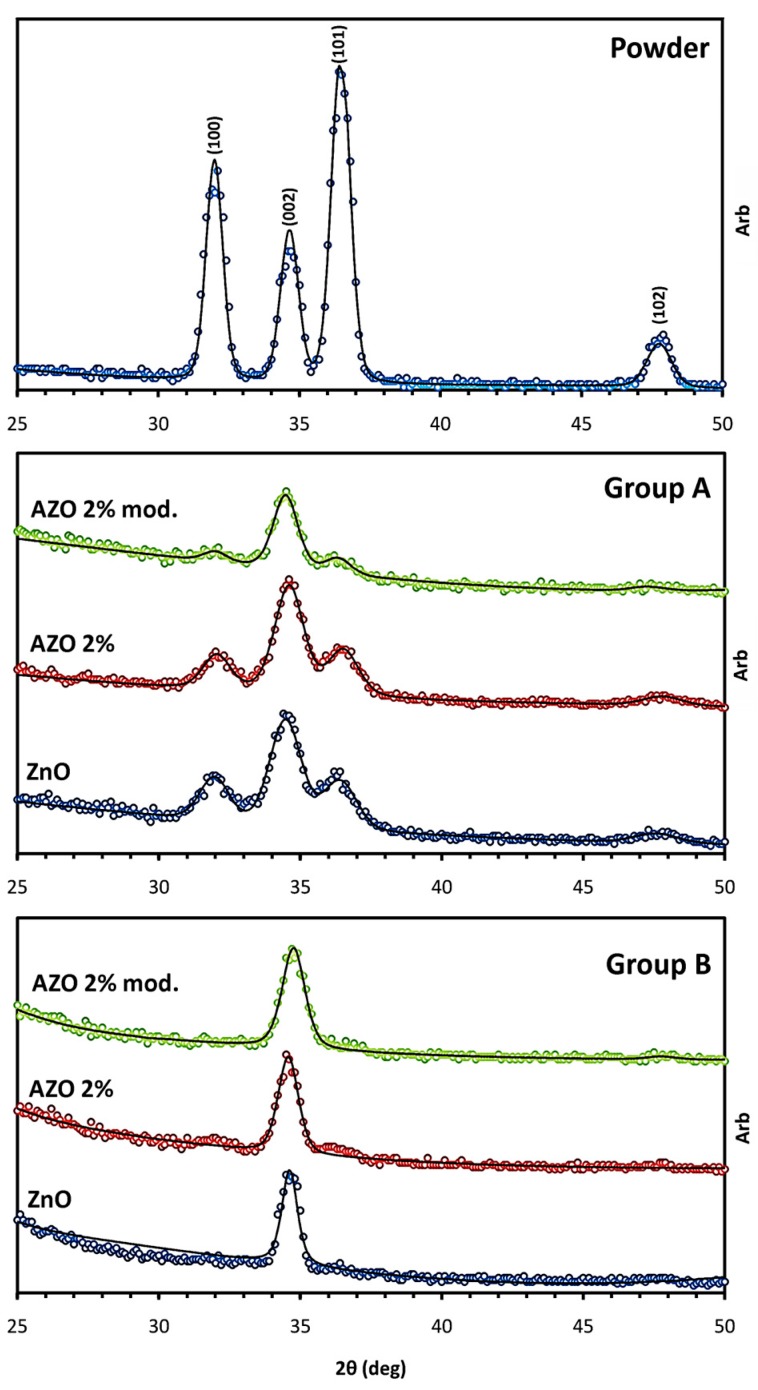
X-ray diffraction patterns for the powder particles and Group A and Group B samples after the final heat treatment.

In Figure 4, the XRD patterns concerning the samples of both groups after the final heat treatment are displayed in addition to the spectrum of the calcined powder. By performing the curve fitting on the peaks through the MAUD software, the thin films’ microstructural parameters were evaluated, as shown in the Table 3. The texture coefficient of the (002) peak, T_c_(002), was also calculated for each sample, using Equation (1), in order to quantify the structural monoorientation toward this plane:

(1) $$T_{c}\left(002\right)=\frac{\left[I\left(002\right)/I_{p}\left(002\right)\right]}{\frac{1}{n}\cdot \sum \left[I\left(\mathrm{hkl}\right)/I_{p}\left(\mathrm{hkl}\right)\right]}$$

where I(hkl)/I_p_(hkl) denotes the ratio of the (hkl) peak intensity in the textured sample to the one in the randomly oriented pattern (powder); n is the number of considered reflections; and Σ[I(hkl)/I_p_(hkl)] indicates the summation of ratios for all the n reflections [26]. The texture coefficient of planes in the powder pattern is T_c_(hkl) = 1; and any deviation in films’ patterns as T_c_(hkl) >1 or T_c_(hkl) < 1 implies a preferred growth, as an abundance or scarcity of grains oriented in the related direction, respectively [27]. Moreover, the relative intensity of the (002) peak, I_r_(002), was calculated as the ratio of the (002) peak intensity to the summation of all the reflections’ intensities [28].

For the evaluation of electrical properties, the four-point probe test was performed on the samples after the final and additional heat treatments. The results in the form of sheet resistance, R_sh_, and resistivity, *ρ*, are reported in Table 4. R_sh_ values, averaged on four different measurements, were estimated using Equation (2) as:

(2) $$R_{\mathrm{sh}}=\left(\frac{\pi }{\ln2}\right)\frac{\Delta V}{I}$$

in which ΔV is the potential difference between the voltage probes, while the constant current flow of I is induced to the sample via the current probes. The ratio is multiplied by correction factor for thin films [29]. Then, *ρ* values were obtained by multiplying R_sh_ to the film thickness value. The degree of uncertainty is difficult to quantify, but the deviation was considered mainly due to systematic errors resulting from the probes’ surface area and their non-ohmic contacts, which affects the relative behavior of different films in a similar way [30]. The thickness of the films was determined as d = 106 ± 2 nm through spectroscopic ellipsometry using a two-layer model by the DeltaPsi2 software. It assumes that the film consists of a main part as the first layer, and the top layer with surface roughness was considered to be a combination of the film and voids containing air. Then, the film thickness was estimated as the summation of both layers. Compared with the one-layer model, this model resulted in a high fitting quality of around 0.9. The optical properties of the substrates were obtained from tabulated data.

**Figure 5:**
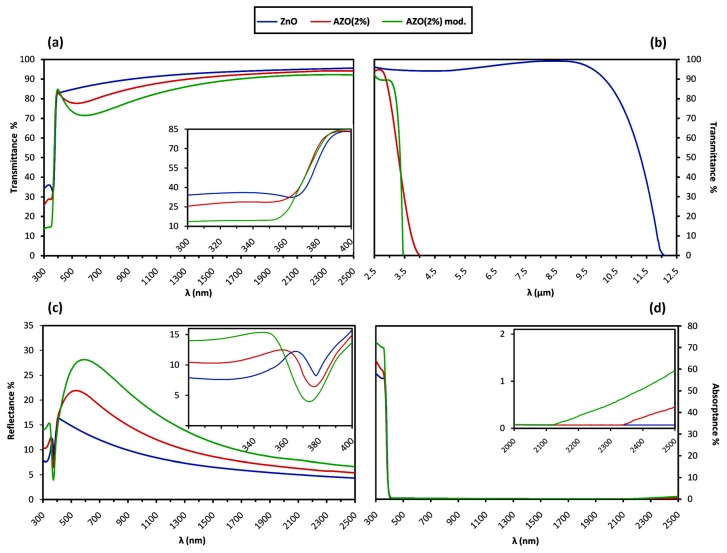
UV-Vis-NIR spectra of Group B samples: (**a**) transmittance, (**b**) extended transmittance, (**c**) reflectance, and (**d**) absorptance. Inset graphs for (**a**,**b**) show the magnification around the absorption edge, while for the absorptance spectra, the inset is the magnification of the far right range.

The optical transmittance T and reflectance R spectra of Group B samples after the additional heat treatment are compared in Figure 5. An extended spectrum in the IR range is included for the transmittance as well. Since the measured values of T and R are affected by the substrate, the following equations were used to estimate the films’ absolute values, supposing that the substrate is homogenous and transparent with negligible absorption [31]:

(3) $$\{\begin{array}{l} T=\frac{1+R_{0}-2\Phi_{R}{\left(R_{0}\right)}^{2}}{{\left(1+R_{0}\right)}^{2}-{\left(R_{0}\cdot \Phi_{T}\right)}^{2}}\cdot \Phi_{T}\\ R=\frac{2\left(1+R_{0}\right)\Phi_{R}-{\left(\Phi_{T}\right)}^{2}}{{\left(1+R_{0}\right)}^{2}-{\left(R_{0}\cdot \Phi_{T}\right)}^{2}}\cdot R_{0} \end{array}$$

in which R_0_ is defined by the refractive index of substrate, ns, as $R_{0}=\frac{{\left(1-n_{s}\right)}^{2}}{{\left(1+n_{s}\right)}^{2}}$, and also $\Phi_{R}=\frac{R_{\mathrm{meas}}}{R_{s}}$ and $\Phi_{T}=\frac{T_{\mathrm{meas}}}{T_{s}}$ represent the ratio of the measured reflectance and transmittance of the sample to the ones of the bare substrate, respectively. Having the absolute values of T and R, the absorptance A is obtained as A% = 100 − T% − R%. The average values of spectrophotometry measurement within the visible range from 400 to 700 nm are reported for all the samples in Table 5 as $\bar{T}\%$, $\bar{R}\%$, and $\bar{A}\%$.

The absorption coefficient α as a function of T and R values and the film thickness d is given by the following equation, which takes the multiple internal reflections into account [32]:

(4) $$\alpha =\frac{1}{d}\ln\left[\frac{\left(1-R^{2}\right)}{2T}+\sqrt{\frac{1-R}{4T^{2}}+R^{2}}\right]$$

Having α, the optical band-gap energy E_g_ was estimated from the conventional Tauc-plot method. According to the Tauc empirical rule [33], for the incident photons with energy levels higher than E_g_, which are identified by the fundamental absorption, α is a function of photon energy E = hν with the below equation for the direct transition occurring in the ZnO band structure:

(5) $$\alpha \cdot h\nu \;\sim \;{\left(h\nu -E_{g}\right)}^{0.5}$$

**Figure 6:**
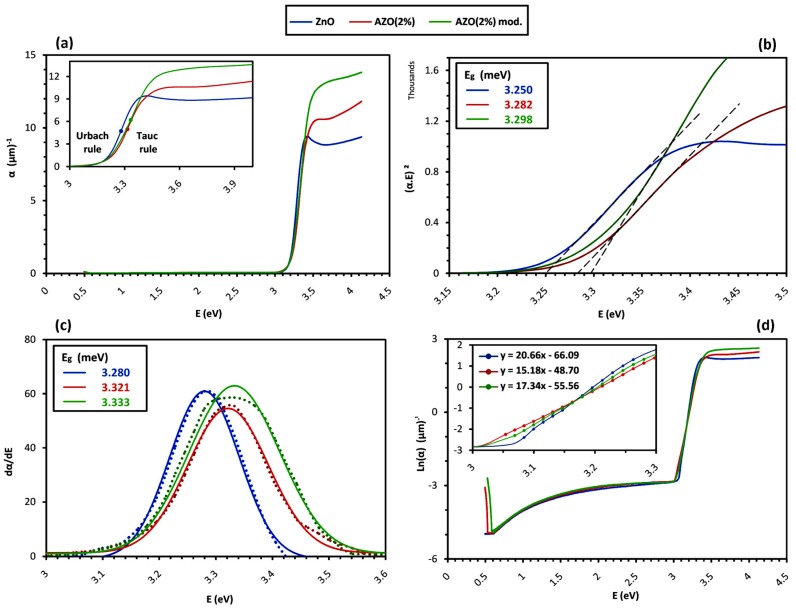
(**a**) The plot of α (μm^−1^) versus hν. The inset is the magnification of the high-energy part, and shows the regions governed by Tauc and Urbach rules. The band-gap energies are marked with solid dots. (**b**) Determination of the band-gap energies via the Tauc-plot method; and (**c**) the alternative method to find the band-gap using the maximum of the dα/d(hν) curve (dotted curves). The solid curves show the Gaussian fits for dα/d(hν) curves. (**d**) The plot of Ln(α) (μm^−1^) versus hν. The inset shows the magnification of the linear parts. The slopes indicate E_u_ values reciprocally.

So, the extrapolation of the linear part of (α·hν)^2^ versus hν gives the approximate E_g_ value at the hν axis, as illustrated in Figure 6b. However, this equation assumes an ideal parabolic band structure, and it is reported that in the case of broadening the fundamental absorption edge as a result of doping or structural non-uniformities, the Tauc-plot method may underestimate E_g_ [34,35]. Therefore, a corrected value of E_g_ was determined by an alternative method using the maximum of the first derivative of the absorption coefficient as the function of photon energy, dα/d(hν), as shown by dotted lines in Figure 6c [36]. All the curves follow the Gaussian trend, which is depicted with solid fitting lines. The difference between the obtained values for the band-gap energy ΔE_g_ is associated with the degree of absorption edge broadening, and is shown to be related to the damping energy Γ by ΔE_g_ = (π/4) Γ [33]. The E_g_ values calculated by both methods in addition to the Γ values are reported in Table 5.

**Figure 7:**
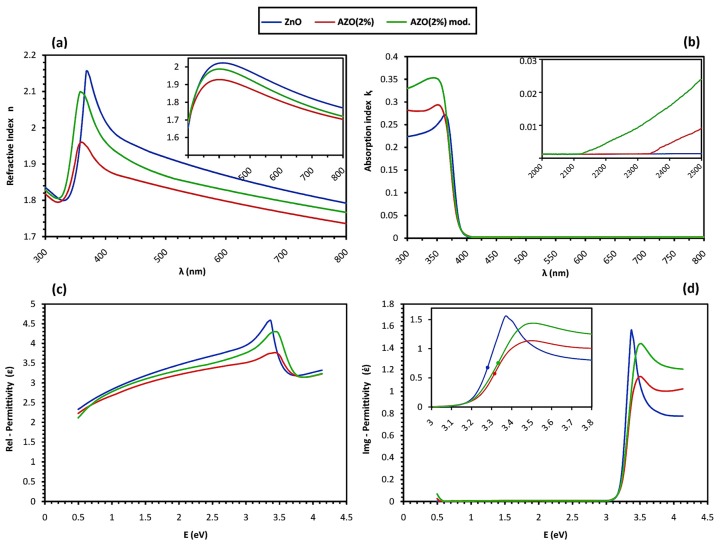
Calculated optical parameters of the films; (**a**,**b**) are the refractive index n and the absorption index ᶄ as functions of the wavelength, respectively. The inset shows n values fitted to the Cauchy dispersion formula. (**c**,**d**) are the real part and imaginary part of the relative permittivity, ε and ἐ, as functions of photon energy, respectively. The inset shows the magnified part of ἐ around the absorption edge, and the band-gap energies are marked out with solid dots.

For the incident photons with the energy just below the band-gap energy, α shows another form of dependency on hν, as stated by the Urbach empirical rule [37]:

(6) $$\alpha \;\sim \;\exp\;\left(h\nu /E_{u}\right)$$

in which E_u_ is the Urbach energy, corresponding to the width of the absorption edge below the band-gap [38]. By plotting Ln(α) versus hν, the value of E_u_ is calculated by taking the reciprocal of the slope of the linear part in the lower photon energy region of the curve, as depicted in the inset of Figure 7b. The obtained values of E_u_ are reported in Table 5.

Afterwards, the optical parameters were calculated including the refraction function as $\bar{n}=n+iⱪ$ and the relative permittivity function as $\bar{\varepsilon }=\varepsilon +iἐ$; where the absorption index ᶄ, refractive index n, and real and imaginary parts of relative permittivity, ε and ἐ, were obtained via the relations below:

(7) $$\{\begin{array}{c} ⱪ=\frac{\alpha \lambda }{4\pi }\;R=\frac{{\left(n-1\right)}^{2}+{ⱪ}^{2}}{{\left(n+1\right)}^{2}+{ⱪ}^{2}}\\ \varepsilon =n^{2}-{ⱪ}^{2}\;ἐ=2nⱪ \end{array}$$

Then, n values as a function of λ were fitted to the Cauchy dispersion formula [39]. The result is illustrated in Figure 7. The n values at λ = 450 nm were used to estimate the porosity p of films through the Lorentz–Lorentz equation [40,41]:

(8) $$p=1-\frac{\left(n^{2}-1\right)/\left(n^{2}+2\right)}{\left(n_{B}^{2}-1\right)/\left(n_{B}^{2}+2\right)}$$

Here, we consider that n and $n_{B}$ are refractive index values of the film and of the pure ZnO, bulk respectively. The estimated values for n and p% are listed in Table 5.

As the final step, the FoM values of the Group B samples were calculated. Historically, the first successful suggested definition of FoM was reported by Haacke as FoM = T^10^/R_sh_ [43], in which the dimension is Ω^−1^, and a larger value indicates a better performance. With a similar dimension, a more practical definition was proposed by Jain and Kulshreshtha [3,44], which evaluates the film performance independent of the thickness:

(9) $$\mathrm{FoM}=-{\left[R_{\mathrm{sh}}\cdot \mathrm{Ln}\left(T\right)\right]}^{-1}$$

There is also a more sophisticated definition proposed by Gruner and modified by Coleman relying on a relationship in which T and R_sh_ are correlated to σ_DC_/σ_OP_ as the ratio of dc conductivity to the optical conductivity [45,46]. The derived equation [47] with a similar dimension to Haacke’s FoM is:

(10) $$\mathrm{FoM}=188.5{\left[R_{\mathrm{sh}}\cdot \left(T^{-0.5}-1\right)\right]}^{-1}$$

So, using the data reported in Table 4 and Table 5 about R_sh_ and $\bar{T}$ values and Equations (9) and (10), the FoM values of the Group B samples after the additional heat-treatment films were obtained and displayed in Table 6.

## 4. Discussion

The electrical and optical properties of the obtained films are associated with the preferred crystallization orientation and grain-boundary characteristics of their microstructures [10]. These features are correlated with the both (1) sol chemistry, as it determines the nature and amount of the species in the amorphous deposited layer; and (2) the thermal treatment procedure, which induces the nucleation and crystal growth as well as the decomposition and the release of organic molecules. Generally, the oriented crystallization toward the c-axis is energetically preferred in ZnO thin films over the substrate [16]. Based on the Ohyama proposed explanation [16], the preferred orientation toward the c-axis is even enhanced in case of less overlapping and the coincidence of steps of this sequence: (a) evaporation of the liquid phase and decomposition of the organic residue, and (b) crystallization of the oxide film. Since the structural relaxation of the gel, as a prerequisite for the oxide film crystallization, originates from the evaporation of the liquid phase and decomposition of the organic residue, the better separation of steps helps avoid any deterioration of crystallization uniformity. Such a separation is taken into account by performing the first step during intermediate heat treatments and postponing the second one to the final heat treatment. It requires knowing the physical and chemical properties of the sol species such as the boiling temperature and molecular bonding, in addition to the gel reaction to high temperature. So, a detailed study on the sol chemical evolution as well as the thermal evolution of the gel is taken into consideration, followed by the consequent effects of different sol chemistry and thermal treatment procedures on the microstructural features. Finally, the different optoelectrical behavior arising from the modified microstructure is compared with those of others and evaluated by the FoM values.

### 4.1. Evolution of the Sol

While the commonly used sol–gel routes to synthesize metal oxides are based on hydrolysis reactions of metal alkoxides or their inorganic salts in organic solvents or aqueous media respectively [48], for ZnO-based compounds, using ZAD as an organic salt in an alcoholic solvent has been reported more often, which is an intermediate between the two conventional sol–gel routes. In such conditions, an in-situ formation of alkoxide-based or hydroxide-based compounds happens initially; then, they transform into metal oxide nanoparticles via hydrolysis and condensation reactions [49]. 

The experiment in this study starts by adding 2-ME to the ZAD precursor, which results in the formation of zinc monoacetate, Zn(OAc) [50]; meanwhile, the expected zinc alkoxide is inhibited from forming, since the solubility of simple zinc alkoxides in alcoholic media is restricted to just long-chain alcohols such as oleyl alcohol [51,52]. Then, the supposedly released water molecules of ZAD start to hydrolyze Zn(OAc), forming zinc hydroxide, Zn(OH)_2_, which will undergo condensation to form ZnO during the forthcoming thermal treatment. However, due to the low initial amount of water supplied by ZAD, the rate of assumed hydrolysis is low [53]. The addition of water in this step is not helpful and should be avoided; otherwise, since the solubility of Zn(OH)_2_ in alcohol is limited, white solid Zn(OH)_2_ precipitates [54]. Moreover, the limited solubility in alcohol applies to ZAD as well, because the coulombic hydration sheath surrounding a zinc cation remains attached with it inside non-polar solvents and prevents its dissolution [51,55]. Consequently, all the reactions develop only very partially, resulting in a turbid grayish solution. Eventually, any hydrolysis through the aqueous route is considerably insignificant.

**Figure 8:**
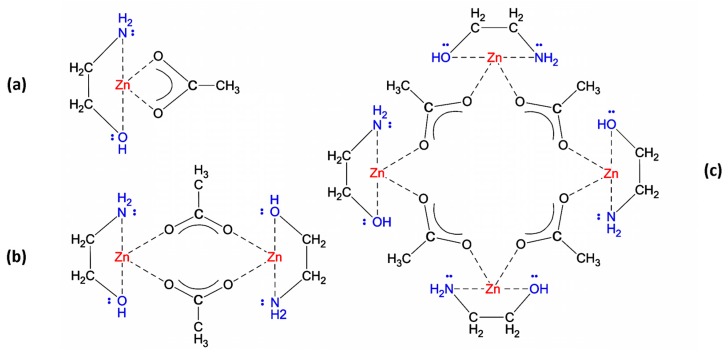
Proposed structural formula of the complex compound in (**a**) a monomer, (**b**) a dimer, and (**c**) tetramer forms.

In order to improve the solubility, it is necessary to introduce an additive compound such as monoethanolamine (MEA), which acts as a nucleophilic agent toward Zn ions. MEA has two Lewis base groups, a hydroxy and an amine, and it is capable of making a bidentate ligand, whether as a chelate to one ion or a bridge between two Zn ions [56]. However, a chelating ligand is expected to be more stable [57,58]. In a similar way, in ZAD and Zn(OAc), acetate is a Lewis base, and a chelating ligand exists between the acetate oxygen atoms and Zn ion, while water molecules form a hydration sheath around the metal core [59,60]. It is reported that by adding amine-containing compounds, the hydration sheath is disturbed, and water molecules are released [55]. Moreover, instead of a selective coordination and ligand exchange with the acetate anion, an additional coordination happens [61]. Therefore, the MEA molecule alongside the acetate ion forms a complex ion cooperatively, in which the central metal ion is coordinated by two chelating ligands: a metal–oxygen core formed by the acetate ion, covered by an organic shell made by MEA. The solubility of the new complex compound, referred to as [MEA][Zn(OAc)], in the non-polar solvent of 2-ME is much more than that before the addition of MEA; so, a clear transparent solution is obtained. However, based on DFT calculations, such a mononuclear compound is not thermodynamically stable in the solution, and a dimer structure, [MEA]_2_[Zn(OAc)]_2_, has nearly 50 kcal·mol^−1^ free energy less than two monomers [62]. In the solid phase, the most stable compound has a tetramer structure, [MEA]_4_[Zn(OAc)]_4_, which is formed by the union of two dimers [63]. Figure 8 illustrates the structural formula related to each one.

As depicted in Figure 8, in the monomer structure, likewise ZAD, there is a chelating ligand between any acetate and Zn ions; while in dimer and tetramer structures, each acetate anion forms a bridge ligand between two metal ions. Similar results regarding the bonding condition were reported through NMR analysis as well [62].

The subsequent transformation of the sol species depends on the competition for the Zn Lewis acid center between nucleophilic species of (–NH_2_) and (CH_3_COO)^−^ as the capping agents, and (OH)^−^ as the hydrolysis agent [53,64]. Considering a fixed amount for acetate anions, it is possible to modify the sol evolution by changing the molar ratio of the other species insofar as the solution remains clear and in equilibrium condition.

The presence of free (OH)^−^ primarily originates from the basic environment. Owing to [H_2_O/Zn^2+^] = 2 in ZAD, the typical initial condition is [OH^−^/Zn^2+^] = 1. However, unlike before the addition of MEA, it is possible to manipulate the ratio by adding a limited amount of extra water, inasmuch as the solution remains clear. The experimented molar ratio of [H_2_O/ZAD] = 2 for additional water increases the amounts of free (OH)^−^ to [OH^−^/Zn^2+^] = 2. It was observed that the addition of more water makes the solution translucent and disturbs the equilibrium. For (–NH_2_), the ratio of [MEA/Zn^2+^] = 1 is extensively used in the literature; however, several researchers have reported that in case of [MEA/Zn^2+^] = 2, a more enhanced texture orientation toward the c-axis direction of the zincite structure [15,53,65] is obtained, as well as a finer crystallite size with less porosity [66]. This approach is also confirmed by the investigations exclusively devoted to studying the effect of the amount of amino additives on the microstructural features of ZnO thin films [67,68,69]. While an equimolar ratio of [MEA/Zn^2+^] is enough to form the [MEA][Zn(OAc)] spices, a higher ratio increases the solution’s pH value, which affects the formation of ionic zinc complexes. It is known that the stable ionic form of Zn in the solution varies by changing the pH; from Zn^2+^ in acidic conditions to a non-ionic state when 6 < pH < 8, and to Zn^2−^ in highly basic condition of pH >12 [70]. The latter pH value, which can facilitate the formation of a stable hydroxide-based complex ion, was obtained by [MEA/Zn^2+^] = 2 in the present study.

**Figure 9:**
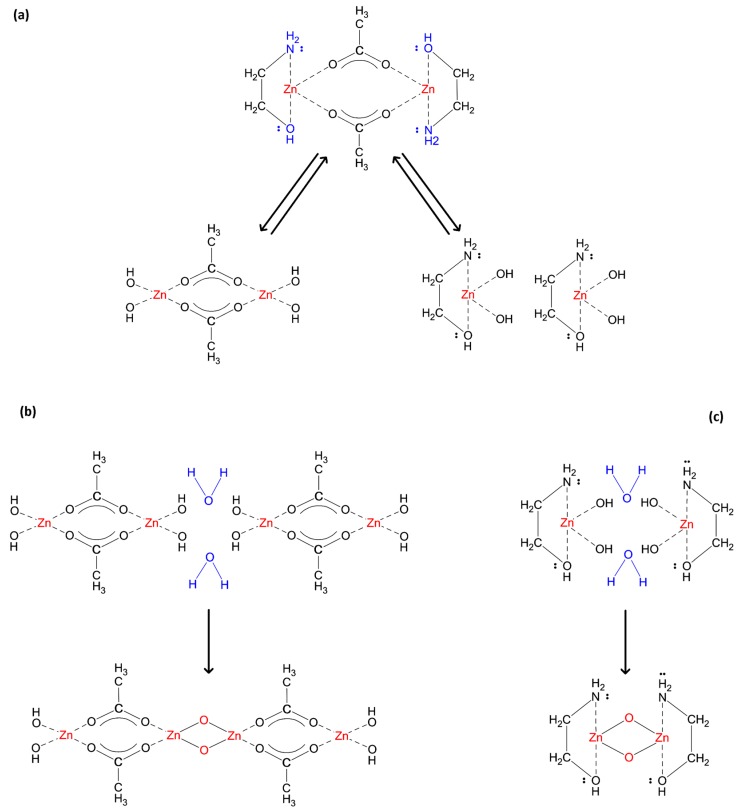
Suggested mechanism for possible hydrolysis (**a**); and condensation reactions (**b**) and (**c**).

Considering the condition obtained from the applied ratios of [OH^−^/Zn^2+^] and [MEA/Zn^2+^], following the addition of extra water, the hydrolysis reaction accelerates and continues throughout the reflux time. It originates from attacking the highly nucleophilic (OH)^−^ to the core of the complex, transforming it to a hydroxide-based complex, as depicted in Figure 9a.

During the aging time, the hydrolysis products are in equilibrium with the initial complex, but they may engage in condensation reactions if water molecules are present, as illustrated by Figure 9b,c. It involves linking the hydrolysis products through the formation of metal–oxygen bonds in an oxolation condensation.

### 4.2. The Gel Thermal Evolution

The transformation of the gel layer into the oxide film is a multiple-step process. It involves the formation of metastable intermediates, pyrolysis and the decomposition of organic parts, the initiation and development of a network of metal oxide crystallites by nucleation and growth mechanisms, as well as releasing the residuals outwards. The initial part of such a process occurs during the drying and intermediate heat treatment, which is performed between each layer deposition; the rest happens throughout the final heat treatment within the whole of the layers collectively. The extent to which the transformation develops during the intermediate heat treatments is decisive in the crystallization behavior and the microstructure of the final film [69]. 

In the IR spectrum of the as-prepared gel, in spite of overlapping, it is clear that the absorption peaks (listed in Table 2) follow a simple superposition of MEA and ZAD peaks. As a suitable indication, we can follow the stretching COO peaks of ZAD (the peaks numbered as 9 and 10) and the stretching CH_2_ peaks of MEA (those numbered as 12 and 13) due to their higher intensities in the initial compounds and being relatively less influenced by overlapping. It is observed that while CH_2_ peaks of MEA appear without any shifting in the IR spectrum of the gel, the two stretching COO peaks of ZAD, referred to as ν_symmetric_ and ν_asymmetric_, show a diverging shift in their wavenumbers. In other words, if the wavenumber separation value is defined as Δν = (ν_asymmetric_ − ν_symmetric_), for stretching COO peaks, we have Δν _(ZAD)_ < Δν _(gel)_, as it is clear in the magnified part of Figure 3 in the carboxyl COO bonds’ frame. It is reported that the Δν value is the characteristic of the acetate/metal bonding form, and as a general rule, ∆ν _(chelating)_ < ∆ν _(bridging bidentate)_ ≤ ∆ν _(ionic)_ < ∆ν _(unidentate)_ [60,71,72]. Accordingly, while nearly all the bonds between the acetate groups and the Zn ion in the ZAD structure are chelating ligands, bridging bonds are expected to exist in the gel. It is in accordance with the presence of thermodynamically more stable dimers and tetramers of [MEA]·[Zn(OAc)] with bridging ligands instead of monomers with chelating ligands.

In the IR spectrum of the treated gel at 275 °C, the most noticeable feature is a drastic decrease in the absorption peaks of organic bonds. It shows that the 55% weight loss and the endothermic peak observed from 195 to 275 °C in the TG-DTA graph are related to the decomposition of zinc organic compounds. Compared with the spectrum of the as-prepared gel, the intensity reduction is more evident for the stretching COO peaks of ZAD than the stretching CH_2_ peaks of MEA (in the gel spectrum, a considerable part of the CH_2_ peaks is hidden due to overlapping with the broad OH peak of ZAD). So, it can be pointed out that the compounds containing acetate groups experienced more deterioration by 275 °C. Besides, while the Δν value for the stretching CH_2_ peaks of MEA remained unchanged, it continued the increasing trend for COO peaks, which was probably a result of the formation of unidentate bonds in the decomposed species. The second important feature is the appearance of a Zn–O bond absorption peak that indicates the existence of crystalline ZnO with tetrahedral coordination. It means that the exothermic process that started at around 250 °C and centered at nearly 300 °C could be associated with the AZO crystallization from newly decomposed zinc compounds. Eventually, 275 °C was selected as the lower limit for an intermediate heat-treatment temperature, since it separates the first sequence of decomposition–crystallization.

At 430 °C, the TG analysis shows 10% greater weight loss than that observed at 275 °C, and it is correlated with the ongoing reduction in the intensity of organic bonds’ peaks in the IR spectrum. Since the maximum weight loss obtained through TG analysis is around 70%, a great amount of organic compounds are decomposed by 430 °C, including those containing MEA. Furthermore, the relatively sharper Zn–O bond absorption peak indicates a fair development in AZO crystallization between 300–430 °C. However, the DTA graph does not show the features regarding neither an ongoing decomposition nor a crystallization development. Instead, an almost flat line with a subtle positive slope is observed, which may originate from the overlapping of two different peaks happening simultaneously between 300–430 °C; an endothermic peak related to the decomposition of organic species containing MEA and an exothermic peak associated to AZO crystallization. Following 430 °C, a net exothermic peak shows that the crystallization overcomes the decomposition afterwards. Accordingly, 430 °C was selected as the higher limit for the intermediate heat-treatment temperature, since it separates the second sequence of decomposition–crystallization.

The IR spectrum of 600 °C shows a very sharp peak for the Zn–O bond in tetrahedral coordination in an organic-free background; nevertheless, an extremely weak yet detectable pair of peaks related to the CH_2_ bond of MEA is present. This temperature is associated with 70% weight loss in the TG graph, which remains constant up to the end of analysis at 700 °C. So, 600 °C is a suitable choice for the final heat-treatment temperature.

### 4.3. Structural Analysis of the Films

The X-ray diffraction patterns in Figure 4 together with the extracted data in Table 3 provide a basis for studying the correlation of samples’ microstructures with the sol chemistry and applied thermal treatment through comparing them with the same batch or with the other group samples. Accordingly, the different thermal treatments for the Group A and Group B samples seem to have a determining effect on the texture coefficient T_c_ and relative intensity I_r_ of the (002) peak; however, other parameters, including the crystallite size and lattice strain, are less affected by the thermal treatment. The only other noticeable variation is observed when comparing AZO structures with the undoped ZnO ones; where altering the sol chemistry by the introduction of Al^3+^dopants causes smaller crystallite sizes and increased microstrain values, independently of the applied thermal treatment. This is in accordance with observing a slight broadening of the (002) peaks in AZO patterns. On the other hand, altering the sol chemistry in terms of sol modification seems to have no effect, although the c-axis microstrain values are fairly affected in the modified AZO ones.

In order to discuss the samples’ texture, the powder pattern is considered as the reference. This pattern shows a set of well-defined diffraction peaks concerning a highly crystalline phase with randomly oriented crystallites in the hexagonal zincite structure, according to the JCPDS database (PDF no.36-1451). For the films of Group A, apart from a lower degree of crystallinity with respect to the powder sample, a greater increment is observed for the T_c_(002) and I_r_(002) values, showing a preferred orientation along the c-axis. However, only the Group B samples display a totally mono-oriented structure along the c-axis direction; meanwhile, the intensity of the other peaks is insignificant. At the same time, through comparing the texture values within the groups, it is observed that the different chemistry of the deposited sol does not lead to a significant texture variation. So, it can be concluded that the structural orientation in the post-annealed films is essentially a matter of nucleation and grain growth [73], depending on the thermal treatment procedure. Accordingly, the thermal treatment with a high temperature approach performed on Group B samples at 430 °C and 600 °C is recognized as more appropriate for decreasing the overlapping of decomposition and crystallization, and therefore obtaining a structure with a stronger c-axis preferred orientation. This conclusion could be associated with the TG-DTA measurement result. As mentioned in Section 4.2, the total weight loss of the gel equals nearly 70%. At 275 °C and 430 °C, the weight loss is roughly 55% and 65%, respectively. Thus, performing the intermediate heat treatment at 430 °C compared with 275 °C results in fewer amounts of organic residual remaining to be decomposed during the final heat treatment in which the grain crystallization and growth occur. Consequently, due to less overlapping between the crystallization and decomposition steps, the morphological order of grains follows the energetically preferred columnar orientation during the nucleation and growth [16]. Accordingly, in the nucleation step, the driving force is the reduction of free energy through the transformation of the metastable amorphous phase to the crystalline state; so, the initial orientation of nuclei develops in a way to bring about the minimum free energy configuration. Considering a heterogeneous nucleation at the film/substrate or film/film interface, since the basal plane in the ZnO structure has a lower surface energy [74], the nuclei at which their basal plane is parallel to the substrate surface possess the smallest amount of free energy. Such nuclei orientation can be considered as the origin of preferred orientation along the c-axis [73], which is maintained during the crystal growth as well. The crystal growth step throughout the final heat treatment is also driven energetically based on the difference between the basal and lateral planes. In the hexagonal structure of ZnO, the two basal planes are composed of just O^2−^ or just Zn^2+^ ions; therefore, due to being polar and metastable, they show a greater reactivity tendency to impose less energy to the system, which results in a faster growth rate. On the contrary, the lateral planes are non-polar and electrically neutral with a higher stability. Supposing a full coverage of the substrate, in the initially formed particles, the c axis-oriented growth perpendicular to the substrate dominates, due to the faster development of the polar planes [53] and a dense columnar morphology results. Such energetically preferred columnar morphology for ZnO is not particularly developed by the sol–gel method [16], and is reported to form through other techniques such as sputtering [74]. However, this morphology is obtained via the sol–gel method only when the nucleation occurs at the film/substrate or film/film interface in a heterogeneous manner. If the thickness of each individual layer is larger than the average crystallite size, the homogeneous nucleation also occurs inside the film volume, resulting in a porous granular morphology [75,76,77]. In this study, the obtained final thickness after the deposition of 15 layers is in the range of 106 ± 2 nm, indicating that the thickness of each individual layer could be estimated to be considerably smaller than the average grain size of 20 to 30 nm, as reported in Table 3. Accordingly, a fully heterogeneous nucleation is claimed in the deposition of all the layers for the samples of both groups, which fulfills the prerequisite for obtaining textured films of columnar morphology. Thus, the lower T_c_(002) and I_r_(002) values observed for the Group A samples could be only considered as the negative consequence of more overlapping of the decomposition and crystallization steps, whereby the release of organic residuals disturbs the formation of energetically preferred columnar morphology.

Regarding the c-axis microstrain values, it is observed that the modified AZO structures in both groups show slightly higher values than those of pure AZO, considering that the negative sign only indicates the compressive stress mode. Originally, the film stress comprises two components: an intrinsic part due to point defects and crystal lattice distortions, and an extrinsic part due to the thermal strain related to the different thermal expansion coefficients for the film and the substrate [78]. The latter seems to be insignificant in the c-axis direction; since by assuming the validity of the thin film approximation in having much larger lateral dimensions than those of the thickness, the thermal strain of the film is under a plane stress condition [79]. The intrinsic part mainly results from the substitution of Zn^2+^ by the smaller Al^3+^ ion and/or the presence of oxygen ion vacancies, and therefore, it leads to the unit cell contraction and observing compressive strain. For the undoped ZnO samples, the absence of the dopants could lead to lower unit cell contraction and less compressive strain. For AZO samples, the subtle difference in strain values is likely to be caused by the growth process itself, since the doping levels are identical. Accordingly, the fairly lower strain levels observed for the modified AZO structures could imply the slightly enhanced crystallinity of their structures after the growth process, originating from less crystal lattice distortion and fewer numbers of defects such as grain boundaries and porosities [17].

### 4.4. Electrical Behavior of the Films

Preliminary, the electrical performance of the TCO film directly depends on the concentration N and mobility μ of the charge carriers, which are in turn altered by the microstructural variations. This is confirmed by the result displayed in Table 4 regarding the electrical resistivity of the samples. Accordingly, after the final heat treatment, the resistivity values are around one order of magnitude smaller in the AZO films of Group B compared with those of Group A. This indicates that improved electrical conductivities are obtained through performing the high-temperature approach of thermal treatment, which results in structures with stronger c-axis orientation and greater T_c_(002) and I_r_(002) values. Besides, in both groups, fairly lower resistivity values are detected for the modified AZO films. However, the resistivity of the undoped ZnO sample is not altered by the microstructural improvements.

These correlations could be associated with the polycrystalline nature of the films and the contribution of grain boundaries in the charge carrier transport in terms of degrading the mobility of electrons, and therefore, the conductivity of the films. Generally, the charge carrier transport in doped semiconductors is limited through three independent scattering mechanisms [80,81]: (1) the scattering by the host-lattice vibration, (2) the ionized scattering due to the distortion resulting from the presence of intrinsic and extrinsic carriers, and (3) the scattering caused by surface defects. Higher values of mobility are expected for the undoped ZnO samples, since the ionized scattering is less frequent. This is in contrast to the AZO samples, in which due to the huge numbers of carriers added through Al doping, the charge transport is accompanied by ionized scattering, and therefore, lower mobility values are expected. Among the AZO samples, considering the impeding effect of the host-lattice scattering being small at room temperature [11,81], and an identical ionized scattering due to the equal level of doping, the surface-defect scattering is the only reason for the difference observed in conductivity values. The surface-defect scattering is mainly caused by grain boundaries, and their effect on the electrical conductivity was explained by Seto [10] through defining the barrier model. Accordingly, the crystallographically disturbed surfaces between the grains contain a high density of defects and impose a surface-defect energy E_s_ to the structure as a direct function of the density and the surface energy of the grain boundaries. With respect to the electronic states, E_s_ is interpreted as localized electronic states within the band-gap with a density value correlated to the defects’ density. Since the Fermi level is above the localized states in n-type semiconductors [11,82], the localized states related to grain boundaries are acceptors for the adjacent grains and trap charge carriers from the bulk of the grains. It leads to forming (1) charged boundary surfaces, and also (2) the so-called charge “depletion region” in the bulk of the grains near the boundaries and along them. The charged boundaries establish a potential barrier that impedes the charge transport across the grain boundaries and also inside the bulk along the depletion regions [80,81]. Consequently, the non-affected volume for high-mobility charge transport is expected to be larger for a microstructure with larger grains and a narrower depletion region [83].

This explanation justifies the increase in the conductivity values of AZO samples in Group B compared with those of Group A after the final heat treatment. Since among the structures with relatively equal crystallite size, a mono-oriented structure contains a lower density of grain boundaries, the higher (002) texture coefficients could bring about less restriction for charge transport by forming narrower depletion regions, and therefore higher values of carrier mobility in the Group B AZO samples. Moreover, in modified AZO films, the comparatively higher conductivity could be associated to moderately larger crystallite sizes and less microstrain values. However, for undoped ZnO samples, the absence of adequate charge carrier concentration is the predominant reason for high resistivity, making the grain-boundary effect by far insignificant.

Another point concluded from Table 4 is the significant effect of the thermal treatment atmosphere that is observed as the result of additional heat treatment. Accordingly, the resistivity values of the Group B samples decrease by nearly two orders of magnitude, even for the undoped ZnO sample, which implies a noticeable change in the electronic band structure beyond the microstructural variations.

Together with the extrinsic carriers imported to the ZnO lattice by Al^3+^ dopants, another part of the carrier concentration is intrinsically supplied by the lattice point defects, including oxygen vacancies V_O_ and interstitial zinc ions Zn_i_ [84]. While V_O_ and Zn_i_ act as donor states and are considered as the origin of n-type conductivity in undoped ZnO, the zinc ion vacancies V_Zn_ and the interstitial oxygen ions O_i_ act as acceptor states against the intrinsic conductivity. V_O_ is by far the most abundant point defect due to its much lower formation energy [85,86]; however, performing the final heat treatment in air atmosphere promotes the chemisorption of acceptor oxygen molecules on the film surface, inside the pores, and between the grain boundaries. Similar to O_i_, the oxygen chemisorption involves capturing electrons from the ZnO bulk located in the conduction band and the formation of O^−2^ and O^−^ ions, leading to the carrier concentration loss. Moreover, it helps the charge depletion regions in the bulk, which degrades the mobility of the carriers, as mentioned above [87]. Hence, the low partial pressure of oxygen during the additional heat treatment could enhance both the concentration and mobility of carriers by giving rise to the better desorption of oxygen from the structure [88,89]. By partially introducing H_2_ to the furnace, the additional heat treatment continues under the reducing atmosphere. It is reported that [90] hydrogen treatment increases the ZnO intrinsic conductivity through accelerating the oxygen desorption and also by the etching of small grains that grow among the larger ones, leading to reduced grain-boundary scattering. More importantly, hydrogen behaves as a shallow donor in the electronic band structure of ZnO [91], leading to a charge carrier increase as well. These explanations justify the functionality of the low oxygen pressure and also the role of hydrogen treatment in decreasing the intrinsic resistivity of the undoped ZnO sample of Group B by performing the additional heat treatment.

For the AZO samples, the effectiveness of the additional heat treatment is beyond the advantages mentioned for the undoped ZnO sample and expanded to alter the condition of extrinsic carriers as well, regarding the position of Al^3+^ ions in the ZnO lattice. In the hexagonal structure of ZnO, the O^−2^ and Zn^2+^ ions occupy the tetrahedral positions, while all of the octahedral holes are empty, providing suitable space for interstitial Al^3+^. However, ideally, and in order to add a free electron to the lattice, one Al^3+^ must substitute for one Zn^2+^ at the tetrahedral position, since in the case of the interstitial occupation of octahedral sites, it behaves as an acceptor and decreases the conductivity [92]. In an inclusive investigation through performing ^27^Al NMR spectrometry on AZO powders [93], Damm et al. reported that while before the reductive annealing, the relative occupancy of Al^3+^ in octahedral positions is more or less equal to the tetrahedral ones, after the reduction, annealing a dominant substitutional tetrahedral occupancy of Al^3+^ is observed. It was also confirmed by Momot et al. that upon the reductive annealing, a rearrangement of the Al^3+^ coordination in the ZnO lattice happens by the migration of the Al^3+^ ions at interstitial positions to the substitutional positions [94]. This “dopant activation” contributes to boosting the conductivity up through an increment of the active charge carriers.

### 4.5. Optical Behavior of the Films

Based on Maxwell’s equations, the interaction between a medium and the incident electromagnetic wave depends on the electrical and magnetic characteristics of the medium; or more precisely, on the electrical conductivity σ and permittivity ε as well as the magnetic permeability [95]. So, regarding non-magnetic compounds with permeability of the unit of value, including conventional TCOs, the optical behavior depends on whether the medium shows dielectric or conductive features. The distinction is that only bound electrons exist in the former, while in the latter, moving free electrons as the charge carriers respond to the incident wave as well. This interaction depends on the concentration and mobility values of free electrons, indicating that different optical behaviors are expected for samples of a certain compound with different N and μ values, and hence, different conductivity. Such correlation between the optical and electrical behaviors is confirmed by the results displayed in Table 5 as the summary of the observed values in Figure 5, Figure 6 and Figure 7 for the optical properties of Group B samples after the additional heat treatment. In the following, we discuss the effect of N and μ on the observed optical behavior in two forms of optical absorption and dispersion.

#### 4.5.1. Absorption Behavior

For high-energy photons of around 3 eV and above (equivalent with UV wavelengths of λ ≤400 nm), the sharp reduction in the transmittance spectra in Figure 5a shows that the optical behaviors of the samples are dominated by the fundamental absorption occurring as an excitation of electrons. In this range, as depicted in the inset of Figure 6a, the absorption coefficient α shows two different relationships with the photon energy:

The first one, which was stated as the Tauc empirical rule in Equation (7), is a parabolic relationship applied to the incident photons with energy levels higher than the band-gap energy (E_g_ < E); here, the eventual excitation of electrons happens as a direct “interband transition” from the valence to the conduction band. The E_g_ value for the band structure of a pure undoped ZnO single crystal has been reported controversially ranging from 3.1 to 3.4 eV; however, values above 3.3 eV are confirmed more frequently [34,79]. This is higher than the calculated E_g_ value of the undoped ZnO sample, which was reported as around 3.28 eV in Table 5. This “band-gap narrowing” is in accordance with previously reported data about ZnO thin films prepared by the sol–gel method on the quartz substrate [41,79], and it is associated with the existence of surface defects. As mentioned in Section 4.4, due to being of small grain size, there is a high density of surface defects in the form of grain boundaries that create localized states within the band-gap and trap the charge carriers from the bulk of the grains. It is reported that charged boundary surfaces decrease the E_g_ value [41,79]. So, the absorption coefficient and E_g_ value of the undoped ZnO thin film sample are smaller than those of a single-crystal ZnO. On the other hand, there is an increase in the E_g_ values of AZO samples compared to the undoped ZnO one. This “band-gap widening” is reported to occur proportionally with increasing the free charge carriers through importing Al^3+^ dopants to the ZnO lattice [87]. As stated in Section 4.3, the substitution of smaller Al^3+^ with Zn^2+^ ions increases the compressive strain along the c-axis direction of the AZO samples, which is reported to cause band-gap widening compared with the undoped ZnO sample [78,79,96]. More importantly and based on the Moss–Burstein effect, doping generates donor levels at the base of the conduction band and increases the charge carriers through filling them with free electrons. Hereafter, the excited electrons from the valence band must overcome an additional energy gap to reach empty available states [36]. So, a higher widening is expected for a larger concentration of free charge carriers N. Based on the differences in the E_g_ values of the Group B samples, we can conclude that N follows this trend: N_(ZnO)_ ≪ N_(AZO 2%)_ ≤ N_(AZO 2% mod.)_.

At the same time, the second relationship is an exponential one, which is introduced as the Urbach empirical rule in Equation (8) for the photons with energy levels of E < E_g_. It describes the transition of electrons in the localized states positioned within the band-gap and indicates the absorption of photon with energy levels even below the band-gap energy. The Urbach energy E_U_ characterizes the degree of absorption edge extension into the sub-gap region and is related to the crystalline lattice disordering caused by the thermal vibrations and crystallographic faults [38]. Thus, in a constant temperature, structural defects in the form of deviation from the perfect periodicity of an ideal crystalline state have been the main contribution to increasing the width of the absorption edge and observing higher Eu values [97]. With respect to electronic states, as mentioned in Section 4.4, structural defects introduce localized electronic states within the band-gap, leading to the so-called “tailing” of the states above the valence band and below the conduction band with an exponential distribution [82,98]. Therefore, while the band edges terminate abruptly in a defect-free single-crystalline structure and no optical absorption happens below the band-gap energy, in amorphous or microcrystalline and heavily-doped structures, the localized band-tail states encroaching on the band-gap induce the optical absorption with an exponential dependency on the photon energy [99]. Here, E_U_ corresponds to the width of these localized states, and informs the overall effect of all types of lattice disorders such as strains, dislocations, porosities, and most importantly, grain boundaries that form the trap states together [38,79]. To a minor extent, it is also associated with the fault originating from the remained organic molecules introduced to the system in the role of capping ligands [100], as could be traced in the present study in Figure 3 in the form of a pair of weak peaks related to the CH_2_ bond of MEA after thermal treatment at 600 °C. Since the Fermi level is above the localized states in ZnO [11,82], these states act as electron acceptors and can trap free electrons. So, the charge transport is assumed to be a series of trapping and release events regarding such electron traps. The density of these localized states is a determining factor in the electronic performance of a semiconductor in terms of reducing its charge carrier mobility μ; furthermore, it is found that the density of localized states increases with increasing the E_U_ value [98]. So, based on the differences in the E_U_ values of the Group B samples, we conclude that μ follows this trend: μ_(AZO 2%)_ < μ_(AZO 2% mod.)_ < μ_(__ZnO)_.

In addition to the transition of electrons, the fundamental absorption is also attributed to the formation of excitons. Excitons are bound states between the excited electrons in the conduction band and the corresponding holes in the valence band stabilized through Coulomb force attraction. These excitonic states dominate the absorption above the absorption edge [101]. As observed in Figure 6a, the undoped ZnO sample shows an excitonic peak at around 3.4 eV in the absorption coefficient spectrum α, which is coordinated with the peak at around 364 nm in the absorption index spectrum ᶄ of Figure 7b. The excitonic peak is also correlated with a minimum in the transmittance spectrum in Figure 5a, as a shoulder peak between 360 to 370 nm. However, the excitonic absorption peak is hardly observed for the AZO samples’ spectra, indicating that the so-called “exciton Mott transition” is activated, whereby the interaction of excitons with free electrons in the conduction band alters the electron-hole binding characteristics and results in exciton dissociation, and therefore the broadening or total vanishing of the excitonic absorption peak [102,103,104]. The exciton Mott transition implies the existence of free electrons in the conduction band and a degenerate semiconductor with metallic behavior [35,104]. Here, for both spectra of α and ᶄ, the broadening of the excitonic peak in the modified AZO sample is detected as slightly more intensive, so it may be concluded that a fairly higher density of free electrons in the conduction band of the modified AZO sample causes more exciton dissociation and almost the total vanishing of the peak, and therefore, N_(ZnO)_ ≪ N_(AZO 2%)_ ≤ N_(AZO 2% mod.)_.

For the photons with visible and IR wavelengths of around 400 ≤ λ ≤ 700 nm and λ ≥ 700 nm (possessing the energy levels of 1.6 to 3 eV and below 1.6 eV), the interband transition of bound electrons does not occur, as the incident photons cannot provide the required energy for the electron excitation. So, principally, no absorption behavior is expected in these ranges; however, the presence of free electrons in the conduction band and their response to the incident photons affects the optical behavior of a degenerate semiconductor compared to the non-degenerate one.

In the range of visible wavelengths, as depicted in Figure 5a, the undoped ZnO sample exhibits a high average transmittance up to nearly 90%, while the value drops to roughly 80% for the doped samples. The visible reflectance spectra in Figure 5c also depicts a clear distinction where the average values for the doped samples are nearly 22% and 18% compared to 12% for the undoped ZnO one. On the other hand, the visible range of the absorptance spectra in Figure 5d is almost identical for all the samples, with average values below 1%. Insignificant visible absorption is also confirmed by the spectra of absorption coefficient α and absorption index ᶄ in Figure 6a and Figure 7b, respectively. This observation shows that the transparency loss in the visible range is mainly due to the reflection, and not because of the transition-based absorption of photons.

In the IR range, as depicted in Figure 5a,b, the samples are highly transparent as long as a sharp reduction in the transmittance occurs for all. However, the undoped ZnO sample keeps the transparency for a much broader wavelength interval compared to the doped ones. At the same time, on the far right of the absorptance spectra, as illustrated in the inset of Figure 5d, a new absorption trend of the so-called “free carrier absorption” is observed to initiate only for the doped samples. The free carrier absorption is correlated to the increment of the absorption coefficient for the low-energy photons in Figure 6a,d, and is responsible for transmission loss in the IR range, as illustrated in Figure 5b. The free carrier absorption increases directly with the free carrier concentration N [105,106,107], as the larger value of N changes the onset and maximum wavelength of the absorptance spectrum with a blue shift [3]. The blue shift could be observed for the modified AZO sample in the inset of Figure 5d and also in Figure 5b, supposing that the maximum absorptance happens roughly at the transmission minimum wavelength. Accordingly, through comparing Figure 5b,d it can be concluded that: N_(ZnO)_ ≪ N_(AZO 2%)_ ≤ N_(AZO 2% mod.)_.

#### 4.5.2. Dispersion Behavior

The final argument regarding the samples’ optical behavior could be stated through the dispersion theory, which explains the frequency-dependent response of a medium to the incident wave from the optical and electrical point of view, in terms of refraction and the relative permittivity function. Accordingly, the solid medium is considered as an arrangement of self-oscillating components embedded in a vacuum [108]. Their response to an incident electromagnetic wave is emitting wavelets with the same frequency as that of the incident wave expanding in all directions; however, the wavelets’ interference is constructive just in one direction, and destructive in the other lateral directions. So, a redirected secondary wave is formed and propagates in the medium. The emission of wavelets happens with a delay, so compared to the incident wave, the secondary wave has a phase lag that reduces its amplitude. The phase-lag value of a single wavelet is related to the incident wave frequency ω, and also the medium oscillation resonance ω_r_. The total phase lag is the aggregate of all the phase lags formed by all the components along the propagation path, so it is proportional to the medium thickness. For a thin medium or when the incident wave is of very low energy in which ω ≪ ω_r_, there is almost no delay in wavelet emission, and the total phase lag is nearly zero. In this condition, the secondary wave propagates through the medium with the same amplitude and frequency of the incident wave, which means that the medium is transparent. For a thick medium or when the incident wave is of higher energy, the phase lag increases directly with the ω and thickness. In this condition, the secondary wave has the same frequency, but less amplitude than that of the incident wave, indicating partial energy absorption and less transmission. Finally, for an incident wave in which ω = ω_r_, or when the medium is thicker, the amplitude of the secondary wave becomes zero, and total absorption occurs.

Optically, the redirection of the secondary wave determines the refraction of light and the refractive index value n. The amplitude reduction that resulted from the total phase-lag associates with the absorption index ᶄ, and therefore, the absorption coefficient α, as stated in Equation (9). Figure 7a,b depicts the refraction functions of the Group B samples. The ᶄ value in the visible region is nearly zero for all the samples, following the properties of regular transparent semiconductors. However, in the infrared region as depicted in the inset of Figure 7b, an increasing trend of ᶄ is observed for the doped samples, unlike the undoped ZnO one, indicating free carrier absorption, as mentioned in Section 4.5.1. Similarly, the blue shift of the onset wavelength in the modified AZO sample indicates a slightly higher value of free carrier concentration compared with the pure AZO one. On the other hand, since n is affected by the degree of crystallinity, it is possible to evaluate the density of defects through comparing the n values of the films with those of the bulk. Using the Lorentz–Lorentz relation stated in Equation (10), the porosity volume fraction is estimated as a basis to compare the films’ structural uniformity, and thus, the carrier transport mobility. So, according to the data reported in Table 5, μ follows such a trend: μ_(AZO 2%)_ < μ_(AZO 2% mod.)_ < μ_(ZnO)_.

From the electrical point of view, the response of the medium to the oscillating electric field $\vec{E}$ of an incident electromagnetic wave in the optical frequency range is described by the formation of oscillating electronic dipoles due to a slight shifting of the negative cloud of electrons from positive atomic nuclei. The summation of all the dipole moments is the electronic polarization field $\vec{P}$, which has a phase delay compared to $\vec{E}$, and is related to it through the frequency-dependent parameter of electrical permittivity $\bar{\varepsilon }$ as $\vec{P}\sim \bar{\varepsilon }.\vec{E}$ [108]. Similar to the refraction function, $\bar{\varepsilon }$ is also a complex function and comprises a real part ε, indicating the degree to which the medium can be polarized, and an imaginary component ἐ is associated with the attenuation of an electromagnetic wave passing through the medium [109]. Figure 7c,d compares the permittivity function of the Group B samples. The samples demonstrate an almost a similar trend throughout the selected frequency (energy) region, with a maximum polarization around the band-gap energy as the equivalent for the resonance frequency of dipole oscillation. For the incident $\vec{E}$ of higher frequencies, the dipoles are no longer able to follow the oscillations and the electronic polarization stops, but the dipoles’ oscillation continues until being absorbed and attenuated by the structure. This time-dependent process is known as dielectric relaxation, which is evidenced by a drop in ε and a maximum in ἐ [110]. In spite of a similar trend, it is observed that the undoped ZnO shows sharp peaks for both ε and ἐ spectra around the band-gap energy, while for the doped samples, peak broadening and the reduction of both ε and ἐ values occurs. In ἐ spectra, the undoped ZnO peak correlates with the excitonic absorption, which is broadened for the doped samples under similar justification, as explained in Section 4.5.1. For ε, this observation is attributed to shorter dielectric relaxation, resulting from higher damping intensity against the dipoles’ oscillation [111]. The possible source for the damping of dipoles’ oscillation could be associated with the active electron scattering mechanisms. As stated in Section 4.4, in the undoped ZnO, the ionized scattering is less frequent compared to the doped ones, which is claimed to cause collision-based damping [112]. Therefore, a higher level of damping and shorter relaxation time is concluded for AZO films. Considering the equal level of doping, and thus a nearly identical ionized scattering in AZO structures, the difference in their peak broadening could be related to the damping that originated from the structural non-uniformity of the grain boundaries, which was previously referred to as surface-defect scattering [110]. This means that the lower peak broadening and damping intensity could be associated with less surface-defect scattering in the modified AZO compared with the pure AZO. The effect of grain size reduction on the shortened relaxation time is also observed in other sol–gel derived systems [113]. The proposed comparison among the damping intensity of the samples is in accordance with the calculated values for the damping energy Γ reported in Table 5 as Γ_(__ZnO)_ < Γ_(AZO 2% mod.)_ < Γ_(AZO 2%__)_. Eventually, assuming that $\bar{\varepsilon }$ is affected by the presence of free electrons and structural defects through their impact on the damping intensity, a qualitative estimation for the scattering time τ of electrons could be proposed by comparing the dispersive behavior of $\bar{\varepsilon }$. The scattering time τ, which has an inverse relationship with the damping as τ ~ 1/Γ, is considered to be the average of the time intervals at which an electron in the electric field is accelerated until it collides with other electrons or with structural defects that change its energy. This parameter is fundamentally associated with the concept of electron mobility by a direct relationship as μ~τ [114]. Thus, we can conclude that μ follows such a trend: μ_(AZO 2%)_ < μ_(AZO 2% mod.)_ < μ_(ZnO)_.

## 5. Conclusions

The polycrystalline nature and the grain-boundaries characteristics were determined to be the most crucial factor influencing the electrical and optical properties of sol–gel derived ZnO thin films doped with 2 at.% Al. 

The investigation was conducted to study the effects of sol chemistry and thermal treatment procedures. The sol chemistry was modified by altering the hydrolysis reaction through adding water with the molar ratio of [H_2_O/ZAD] = 2. It was argued that a complex ion forms in the coating sol, in which the Zn^2+^ cores are coordinated by MEA molecules and acetate ions in a dimer structure. The addition of extra water increases the amount of free (OH)^−^ as the hydrolysis agent and accelerates the formation of hydroxide-based complexes owing to the highly basic condition provided by ratio of [MEA/ZAD] = 2. The deposition of thin films was conducted via dip coating 15 layers with a slow withdrawal speed of 2.5 cm·min^−1^ under controlled conditions to obtain the thinnest and most uniform layer after each deposition. The drying and annealing steps were performed under two different low and high-temperature thermal treatment approaches. For the former, 275 °C and 500 °C were selected as the intermediate steps and final cycle temperatures, respectively; for the latter, the selected temperatures were 430 °C and 600 °C, respectively. Moreover, the high-temperature approach was followed by an additional heat-treatment step for 1 h at 400 °C under the reducing atmosphere of Ar/H_2_ flow.

The structural analysis of the films via XRD diffraction analysis showed that the thermal treatment in the high-temperature approach brings about samples with grains of considerably stronger c-axis preferred orientation compared with those obtained from the low-temperature one. This result indicates that the high-temperature approach provides a better separation of the decomposition and crystallization steps during the thermal evolution of the deposited gel; this claim was confirmed through the TG-DTA measurement and FT-IR spectroscopy. Therefore, the energetically preferred columnar morphology with higher T_c_(002) and I_r_(002) values develops less restrictedly through the high-temperature approach, due to being less disturbed by the release of organic residuals during the nucleation and growth of the grains. Moreover, as the second conclusion for this part, the fairly larger crystallite size and slightly lower microstrain values in the modified AZO structures implies an enhanced structural crystallinity after the growth process, originating from less crystal lattice distortion and fewer numbers of defects such as grain boundaries and porosities.

The four-point probe evaluation of thin films’ electrical properties after the final heat treatment showed that the *ρ* values of the doped films were around one order of magnitude smaller through the high-temperature approach compared with the low-temperature one; this was a reduction from around 3 to 4 Ω·cm to less than 0.5 Ω·cm. However, for the undoped ZnO film, the *ρ* value was not affected. Besides, among the doped films, the *ρ* values of the modified AZO films were fairly lower than those of the pure AZO ones. The obtained conclusions confirm the correlation between the grain boundaries’ characteristics and the conductivity values. Providing that enough carrier concentration is supplied via doping, the stronger c-axis-oriented morphology obtained through the high-temperature approach results in a lower density of grain boundaries, which consequently reduces the scattering of surface defects. Therefore, the increase of the carriers’ mobility is believed to improve the conductivity. Moreover, for the modified AZO film, the fairly larger crystallite size and slightly lower microstrain value could provide better mobility and conductivity values compared with the pure AZO ones.

In addition to the high-temperature approach, an additional heat-treatment step performed under the reducing atmosphere of Ar/H_2_ was shown to have an extremely determining effect on the conductivity through decreasing the R_sh_ values by nearly two orders of magnitude: a reduction from around 0.4 to 0.5 Ω·cm to nearly 6 to 15 mΩ·cm.

Finally, the UV-Vis-NIR spectroscopy that was performed to study the absorption and dispersion behaviors of the films supported the higher mobility, and to a lesser extent, the concentration of charge carriers in the modified AZO film. From the results obtained for the Eu values, n values, and from studying the dielectric relaxation, it was concluded that μ_(AZO 2%)_ < μ_(AZO 2% mod.)_ < μ_(ZnO)_. At the same time, by the investigation of the band-gap widening, excitonic transition, and the free carrier absorption in the NIR region, it was concluded that: N_(ZnO)_ ≪ N_(AZO 2%)_ ≤ N_(AZO 2% mod.)_. The obtained orders for N and μ are in accordance with the conductivity order of σ_(ZnO)_ ≪ σ_(AZO 2%)_ < σ_(AZO 2% mod.)_.

Taking the optical transmission into account, the figure-of-merit values showed the following order: FoM_(ZnO)_ ≪ FoM_(AZO 2%)_ < FoM_(AZO 2% mod.)_. This indicates the enhanced performance of the modified film as a transparent conducting film. The result for the modified AZO sample is in the same class of the highest-ranking AZO films previously obtained via sol–gel method, with the resistivity values in the order of few mΩ·cm, which is equivalent with the sheet resistance of R_sh_ < 500 Ω/sq, and the average visible transmittance of $80\%\leq \bar{T}\leq 90\%$ [93]. However, compared to the specifications reported for the AZO films obtained via more sophisticated techniques such as sputtering [115], chemical vapor deposition [116], and atomic layer deposition [117], which provide thin films with resistivity that is one order of magnitude lower and similar visible transmittance, the sol–gel derived AZO films are more suitable where affordable and moderate electrical conductivity is required, such as the inkjet printing of low-cost printed electronics [9].

## Figures and Tables

**Table 1:** The thermal treatment details and thermal history of the samples.

Samples (Substrate)	Intermediate Heat Treatment	Final Heat Treatment	Additional Heat Treatment		
Group A (Soda lime)	Static air at 275 °C for 5 min	Static air at 500 °C for 1 h	None		
Group B (Quartz)	Static air at 430 °C for 5 min	Static air at 600 °C for 1 h	Ar flow at 600 °C for 1 h	Ar/H_2_ flow at 400 °C for 1 h	Cooled down to RT* in Ar/H_2_ flow

* Room temperature.

**Table 2:** Wavenumbers of distinct absorption peaks in the IR spectrum of as-prepared gel, numbered as one to 14 in Figure 3. The molecular bonds and vibration modes of constituent peaks are provided.

Observed Peak (cm^−1^)		Assignments	Observed Peak (cm^−1^)		Assignments
1	622	π (COO) → ZAD	9	1402 *	symmetric stretching (COO) → ZAD scissor deformation (CH_2_) → MEA
2	696	α (COO) → ZAD	10	1577	asymmetric stretching (COO) → ZAD
3	941	out-of-plane deformation (NH_2_) → MEA	11	1593 *	in-plane deformation (NH_2_) → MEA
4	1024 *	rocking (CH_3_) → ZAD stretching (CO) → MEA	12	2852	symmetric stretching (CH_2_) → MEA
5	1072 *	rocking (CH_3_) → ZAD stretching (CN) → MEA	13	2922	asymmetric stretching (CH_2_) → MEA
6	1150	stretching (COH) → MEA	14	3000–3500 *	asymmetric stretching (OH) → MEA asymmetric stretching (OH) → ZAD asymmetric stretching (NH_2_) → MEA symmetric stretching (NH_2_) → MEA
7	1342	symmetric bending (CH_3_) → MEA			
8	1384	symmetric bending (CH_2_) → ZAD symmetric bending (CH_3_) → ZAD			

* Overlap of two or more peaks.

**Table 3:** The results of line profile analysis on the fitted peaks of thin films, as illustrated in Figure 4 for Group A (upper section) and Group B (lower section).

Samples		Crystallite Size (nm)	c-Axis Microstrain (%)	Texture	
				T_c_(002)	I_r_(002)
Group A	ZnO	27 ± 2	−0.12	2.25	0.56
	AZO 2%	20 ± 1	−0.29	2.34	0.58
	AZO 2% mod.	22 ± 2	−0.23	2.94	0.73
Group B	ZnO	29 ± 2	−0.11	3.92	0.98
	AZO 2%	24 ± 1	−0.25	3.84	0.95
	AZO 2% mod.	27 ± 1	−0.19	3.98	0.99

**Table 4:** The results of the four-point probe test on sheet resistance and resistivity values Group A (upper section) and Group B (lower section).

Samples		After Final Heat Treatment		After Additional Heat Treatment	
		R_sh_ (Ω/sq)	*ρ* (Ω·cm)	R_sh_ (Ω/sq)	*ρ* (Ω·cm)
Group A	ZnO	10^6^<	10<	–	–
	AZO 2%	348.6 × 10^3^	3.7 ± 0.3		
	AZO 2% mod.	294.4 × 10^3^	3.2 ± 0.1		
Group B	ZnO	10^6^<	10<	298.5 × 10^3^	3.1 ± 0.2
	AZO 2%	49.7 × 10^3^	0.52 ± 0.2	1.3 × 10^3^	(14.5 ± 0.3) × 10^−3^
	AZO 2% mod.	38.4 × 10^3^	0.41 ± 0.1	543.7	(5.9 ± 0.1) × 10^−3^

**Table 5:** A summary of observed values for the optical properties of Group B samples after the additional heat treatment.

Samples		Spectrophotometry			Band-Gap E_g_ (eV)		Urbach Energy E_u_ (meV)	Damping Energy Γ (meV)	Refractive Index ^*^ n	Porosity ** p (%)
		$\bar{T}\left(\%\right)$	$\bar{R}\left(\%\right)$	$\bar{A}\left(\%\right)$	Tauc plot	dα/d(hν)				
Group B	ZnO	87.7	11.8	0.4	3.25	3.28	48.4	38	2.01	6.4
	AZO 2%	82.3	17.1	0.5	3.28	3.32	65.8	49	1.96	16.2
	AZO 2% mod.	79.1	20.3	0.5	3.29	3.33	57.6	44	1.92	13.5

* at λ = 450 nm from the fitted graph; ** considering n_B_ = 2.12 [42].

**Table 6:** Calculated figure-of-merit (FoM) values for Group B samples after the additional heat treatment through three different definitions.

Samples		Haacke FoM	Jain–Kulshreshtha FoM	Gruner–Coleman FoM
Group B	ZnO	8.61 × 10^−7^	2.46 × 10^−5^	9 × 10^−3^
	AZO 2%	1.07 × 10^−4^	3.9 × 10^−3^	1.4
	AZO 2% mod.	1.72 × 10^−4^	7.7 × 10^−3^	2.75
